# Phosphates in Medical Decoctions and Infusions

**Published:** 1865

**Authors:** 


PHOSPHATES IN MEDICAL DECOCTIONS AND INFUSIONS.—Some interesting experiments made by M. Terreil show that in plants the phosphates exist in a more or less soluble form. If to a filtered infusion of mallows we add a slight excess of acid, or if the same be done to a decoction of taraxacum, the sides of the glass vessel containing the preparation will be found lined with the ammoniaco-magnesian phosphate. Even then all the phosphates are not precipitated, since, if we add sulphate of magnesia, saturated by sal ammoniac, to the filtered liquid, a new deposit of the phosphatic salt will be seen. From this and similar experiments with many vegetable infusions, M. Terreil concludes in the *Bull. de Therap.*, that the alleged superiority of infusion and decoctions in certain cases may be due to the influence of the phosphoric acid and its salts upon the general economy.

